# The Humour and Tragedy of Hospital Life

**Published:** 1922-12

**Authors:** Philip Inman

**Affiliations:** (Secretary-Superintendent, Charing Cross Hospital)


### THE HUMOUR AND TRAGEDY OF HOSPITAL LIFE.

By PHILIP INMAN

(Secretary-Superintendent, Charing Cross Hospital).

A GENERAL hospital, to an eye-witness, is a place of unending fascination and unflagging interest. Within its walls are enacted tragedy, pathos, and even humour. "All the world's a stage," said Shakespeare, but there is little play acting in a hospital. Life is never so real as when it is struggling, ofttimes against heavy odds, for its very existence. Sham and pretence are discarded, and humanity is seen as it really is, and not as it so often appears to be.

In my imagination I can see a large general hospital as I write: A hospital with which I am closely associated. I can see the large airy wards, cheerful and bright, fragrant with flowers :—

"Flowers to these spirits in prison are all they can know of the spring,
They freshen and sweeten the wards like the waft of an angel's wing."

I recall how visitors always admire the shining polished floors, the green-tiled walls, the spotless cleanliness (for in a hospital cleanliness is a necessary adjunct to the re-establishment of health). I visualise the patients in their white-covered beds, ranged down each side of the ward. All are sick, some in pain, but they are enjoying that peace which is part of the cure. I think of the sisters and the nurses, and the remembrance fills me with reverence.

Theirs is a strenuous life—hard work, long hours, small remuneration, nerve-racking and ofttimes depressing scenes, but it is work which only a woman can do, and she excels in it. A leading dramatist once said that anyone who could pass through all the stages of nursing and retain her love for the profession must possess the attributes of an angel. Was it not a wounded soldier who wished to pay a graceful compliment, but unfortunately became rather confused and referred to a matron as the finest fallen angel he had ever seen ?

One has to work in a hospital to understand what self-sacrifice means. I could tell stories of countless silent heroisms, of unselfish service where men and women have given themselves with nothing material to gain, with no self-interest to serve. Let it be remembered that hospital doctors receive no fee or reward for their services, and yet I know of innumerable instances where some of our most renowned physicians and surgeons have forfeited a much-needed night's rest in order to watch by a serious case or perform an urgent operation. One night I chanced to be in the house of one of our surgeons when a messenger arrived from the hospital, asking if he could go and perform an immediate operation. The patient had been taken by the ambulance in a half-starved, destitute condition, was evidently very ill, and an operation was the only hope of saving his life. The night was late, and the wind and rain were beating in fury against the windows. I looked across at the surgeon—one of the giants in the profession. I knew that he had had a long and exacting day. He had told me a few minutes earlier that he had been operating unceasingly since early morning. He had been glad to get home to a cheerful fire and an interesting book. Quietly he listened whilst the message was delivered, then he smiled and said, " Evidently my day's work is not over ! " Hurriedly he put on his coat, and went out without a word of complaint into a cheerless night to help a man in his hour of need. There is not one law for the rich and one for the poor where a doctor is concerned, and I say without fear of contradiction that a servant girl or a labourer receives the same skill and kindly consideration in a hospital from these medical specialists as a peeress or a professor does in the most luxurious and expensive nursing home. A hospital might well be described as the haven of the helpless and suffering humanity can always obtain the passport to its refuge.

In the course of my duties I have witnessed scenes and incidents which often thrilled, sometimes saddened, and occasionally amused.

A short time ago I had a visit from an old woman, a widow evidently from her garb of sorrow, a poor widow as revealed by her faded dress, and I noticed that she carried, almost tenderly, an old-fashioned handbag. It was my Council day, and I did not welcome the intrusion. I waited patiently for her to explain the object of her call, but it was evident she was not to be hurried. Eventually, without answering any of my remarks, she commenced to take from her bag a number of small pill-boxes, which she placed slowly and carefully on my desk. Vacillating between annoyance and amusement, I counted the number of boxes as they were placed, one by one, before me. Thirty, thirty-two, thirty-four, thirty-six, thirty-eight ; then my visitor stopped. The empty handbag was closed again, and she began to speak in a low, pathetic voice : " It is just eight years ago to-day that my husband was brought here as a patient. He was in a bad way, and I was soon told that nothing could be done for him, but you were good to him, and made him very comfortable. His long illness and funeral expenses swallowed up all my savings, but I always wanted to repay you for your kindness, and so I have put by two shillings every week out of my old age pension, and I want to give it to the hospital."

She picked up one of the boxes, and counted its contents into my hand. Then, in the same low voice, she continued, " They all contain the same amount—one pound in two-shilling pieces. It has been a bit of a struggle to come to the hospital to-day, but I felt I must give it now, for I don't expect I shall be here very long." Her voice sounded far away, like one speaking in a dream. I looked at the pile of boxes, and then at the careworn, pale-faced woman before me. I found words at last. " You want to give this money to the hospital ? " I stammered. " I do," she replied, simply. " But can't I do something for you in return ? " I asked. She smiled. " Yes," she said, " there is just one thing that would make me very happy. Will you tell Doctor Garman that John Walker's widow did not forget ! "

Sometimes there is humour even in a hospital. The foibles and fancies to which many patients cling, in spite of advice to the contrary, are really amusing. A woman has attended the out-patients' department regularly for years, although the doctors are convinced there is absolutely nothing wrong with her, and they have tried to make her believe this without success. To tell the woman she looks well is like waving a red flag to a bull. She storms and raves, and is only appeased when she becomes the proud possessor of a bottle of physic. For some time the doctors endeavoured to ease her mind by prescribing for her, but a hospital has to economise, and waste of any kind cannot be tolerated, so one day a bottle of Mist. Rubra (water coloured with cochineal) was given to her. She had complained that the medicine she had been taking had done no good, and this, at any rate, could do no harm. The day of the patient's next visit was awaited with some interest. She arrived in a very excited state, and her first words were, " Oh, please give me another bottle of the mixture I had last week. It has done me so much good ! " When a patient has no organic disease there is much to be said for Professor Coué's *modus operandi*, for this woman has now been " completely cured," and she attributes it all to the wonderful new medicine.

In one of the surgical wards is a Bristol engineer, who has almost reached the allotted span of three-score years and ten. He has been suffering for some time from a malignant growth, and he came into the hospital in search of advice, as he had been told locally that nothing further could be done for him. On his arrival he called at my office, and emphasised the fact that he had come for examination only, and that under no circumstances would he consent to undergo an operation without first going home, and talking it over with his wife. He was placed in a ward for observation purposes, and later in the day the surgeon, an eminent man, came to me and said, "If Evans is operated upon at once, I think I can cure him." I explained to the surgeon the man's attitude. "Yes, I know," he replied. "He will go home and his wife will say, 'If they can't do you any good in Bristol I doubt whether they can in London,' and he will never come back again. You see, I have had so many similar cases. Will you see Evans," he continued, "and persuade him to have it done now?" I agreed. At first and for long the patient was recalcitrant, and flatly refused. Then he asked for time to think about it. I saw he was wavering, and used argument, appeal, persuasion; I told him of remarkable cases where apparently hopeless patients had been cured, that in his case time was valuable, and that I personally had such confidence in the particular surgeon who was attending to him that I would willingly place my life under his care. Then I left him to himself, and when I returned half an hour later he greeted me with the words, "I'm going to risk it." Then, and only then, the full realisation of what I had done came home to me. I went hot and cold all over. What if the operation were not successful? He was an old man; suppose he did not recover? The responsibility I had taken weighed heavily upon me. I could picture a home in Bristol where a woman was waiting for a husband who would never return. I staggered out of the ward. Never shall I forget that night. How I paced the corridor, waiting for the result of that operation! After a time which seemed like eternity the surgeon came out of the operating theatre. I had no need to ask any question; triumph was written on his face.

"How is the patient now?" you ask. Listen to what he says: "Better than I have been for years!" He is going to a convalescent home to-morrow to complete his cure, and in a few weeks he will go home to his wife a new man.

There is a cabinet in our X-ray department which contains all manner of things—pins, thimbles, coins, beads, a lead train, a toy elephant—which people, mostly children, have swallowed, and which have been located by means of the X-ray apparatus. The radiologist tells many amusing stores, amongst them the following: A father sent his boy to buy some cigarettes, but the lad returned a few minutes later, saying that he had been "juggling" with the money, and it had "gone down his throat." The boy was taken to a local chemist, who stated that he could find no trace of the coin, and suggested that the boy had probably spent it and was trying to excuse himself. The father said he would teach his boy to lie to him, and on arriving home proceeded to administer a form of punishment with which many of us were familiar when we were children. After the father had gone to his work the next morning, his mother, as the lad still persisted in his story, brought him to the hospital, where he was X-rayed. The boy had told no lie. The coin was discovered, and later extracted. When the operation was over the lad turned to his mother and said: "I suppose dad can't take back the hiding, as he will get his cigarettes after all!"

One day I was forcibly reminded of the story of the absent-minded girl who, on bidding farewell at the railway station, kissed the porter and gave twopence to her fiancé. All simple powders are given to patients at the hospital in a standardised box. A woman had been given two boxes, one containing boracic powder and the other health salts. On arriving home she saw that her dog had evidently been in a fight, for he was smeared with blood. Knowing that boracic powder was good for bathing wounds, she prepared what she thought was a boracic solution. In her anxiety the lids of the boxes became confused, and the poor dog was bathed with the salts. Fortunately salts, painful though they are when applied to wounds, have good healing properties, and boracic powder, taken as a medicine, would not be so injurious as other powders one might mention. No wonder she complained that "the medicine had not done her a ha'porth of good!"

To many people the most interesting sections of any hospital are the wards devoted to the treatment of children. There is something so tremendously appealing about the helplessness of a child.

The most amazing experience in my hospital life is associated with a boy patient in the children's ward. On a cold December day in 1921, there was brought to us a child of 11 years of age, suffering from a painful and incurable malady—to be precise, cancer. It is a fallacy to think that this dread disease only affects those who are reaching or past middle-age; no one is immune, though happily there are few cases where it develops in children of tender years.

At first it was no doubt sympathy for a child in great pain that caused him to be the object of affection, and it was not until later when we learned to know him that he became the hero of the hospital. Every child has a pet name to the sister in charge of the ward, and this boy was christened "Sonny,"; a more appropriate name could not be found for sunshine radiated from him. Day and night this boy suffered excruciating pain, but in it all and through it all never a murmur or a sob escaped his lips. When things were at their worst, he would bury his head underneath the bedclothes, and as you stood by, you could hear him whistling. Morphia was administered to ease the pain, but after a time it began to lose its effect. Still Sonny remained as calm and bright as ever. Whenever you asked how he was, the reply was always the same, "I feel much better, thank you." A boy, older by some two or three years, happened to be in the next bed, and one night he was so homesick that he cried bitterly for his mother. I turned to Sonny and said, "I have never seen you cry, have I?" "No," he replied, "but sometimes I feel like it!" Some of the happiest hours of my life have been spent by this boy's bedside. I have read to him books of travel and adventure, and his eyes have shone with a new and strange light. He loved stories of heroism, and no wonder, for he himself was of the stuff of which heroes are made.

At Eastertide a remarkable thing happened.

The preacher at a large London church told the story of Sonny, and throughout the whole of his discourse the suffering child was kept before the minds of his congregation. That night Sonny slept peacefully, his first natural sleep for months. He awoke the next morning, refreshed and strengthened, and days followed free from pain, and nights brought their natural refreshing sleep.

A few weeks ago, alas! Sonny took a sudden turn for the worse. Everything possible was done, an operation was performed, radium was administered, drugs were injected, but all of no avail. *And Sonny has passed away.* Right to the end he retained his bright, boyish spirit, and his memory in the Hospital will for ever remain fragrant.

Dr. Wallis, in his book " Travels on the Amazon," makes a very significant statement. He says that as he journeyed into the interior of the country he noticed that the men and women became taller and stouter and stronger until they almost reach the stature of giants.

The explanation is not far to seek; the native does not understand the meaning of the word " hospital." The sick are left to die; the weak have to go to the wall; there is no room for suffering in their scheme of things. English people do not realise how much they owe to the hospitals in our midst. They are like lighthouses, shining above the sea of life, with its currents and cross-currents of sickness, disease and death. It will be a national catastrophe if we ever allow those lights to go out or to burn dim.

